# Pyomyositis in a Patient with IgA Nephropathy and Kidney Transplant

**DOI:** 10.1155/2019/7305683

**Published:** 2019-01-28

**Authors:** M. Xipell, P. Ventura-Aguiar, I. Revuelta, M. Bodro, F. Diekmann

**Affiliations:** ^1^Nephrology and Renal Transplantation Department, Hospital Clinic of Barcelona, Experimental Laboratory in Nephrology and Kidney Transplant (LENIT), Institut d'Investigacions Biomèdiques August Pi i Sunyer (IDIBAPS), University of Barcelona, Spain; ^2^Infectious Diseases Department, Hospital Clinic of Barcelona, IDIBAPS, University of Barcelona, Spain

## Abstract

Infections are among the most common complications transplant physicians face when dealing with solid organ transplant recipients. We present a case of pyomyositis caused by* Staphylococcus aureus *in a patient with IgA nephropathy and a kidney transplant, under treatment with mTOR inhibitors and prednisone. This entity is a rare intramuscular infection, given the resistance of healthy muscle to colonization. We review the most frequent agents, the diagnostic algorithm, and therapeutic alternatives. We also comment on the role of mTOR inhibitors in this case as possible predisposing factor for the infection.

## 1. Introduction

Pyomyositis is a rare intramuscular bacterial infection, given the resistance of healthy muscle to colonization. It presents initially as muscular inflammation, generally of subacute onset, and it later progresses to form abscesses. In an immunosuppressed patient, it may have atypical presentations that could delay its diagnosis or underestimate the signs of severity. We here present a case of pyomyositis in an immunosuppressed kidney transplant recipient.

## 2. Case Presentation

A 54-year-old male with IgA nephropathy had a kidney transplant, with chronic graft dysfunction (basal creatinine 2.7 mg/dL) due to biopsy-proven chronic thrombotic microangiopathy, attributed to tacrolimus. He had no previous history of diabetes, toxic habits, or liver disease, and he was HIV negative. Twelve months after transplantation, he was on treatment with everolimus (trough levels 6-8ng/mL) and prednisone 10 mg/24h.

**Figure 1:**
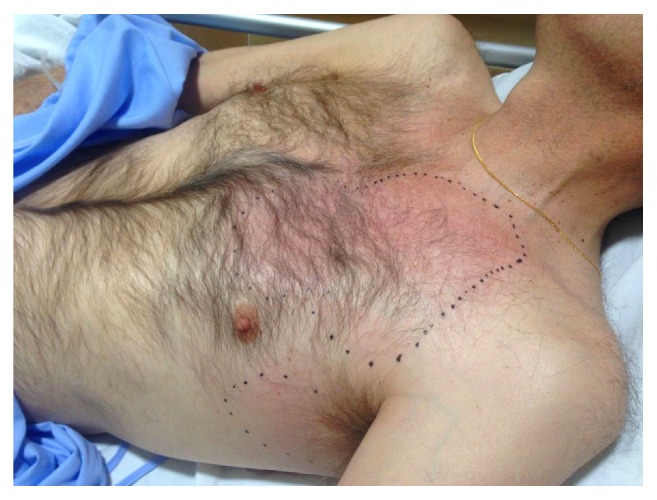
Erythematous plaque in a kidney transplanted patient in the left chest area, slightly painful.

**Figure 2:**
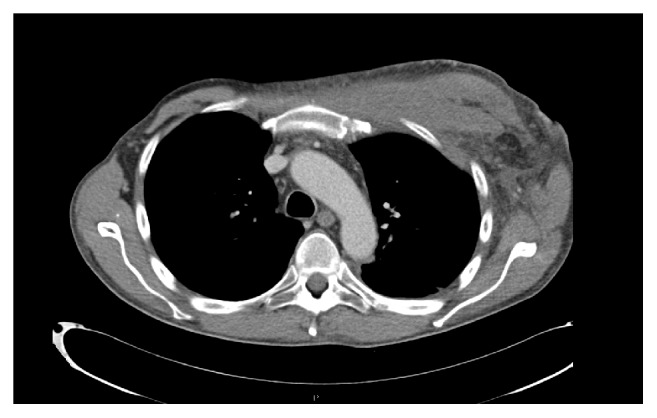
Thoracic Computed Tomography performed in the emergency department.

**Figure 3:**
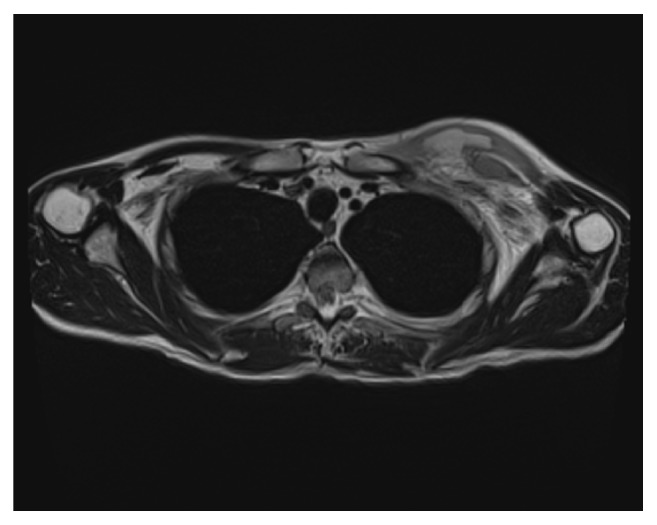
Magnetic resonance image.

The patient consulted in the emergency department due to an erythematous cutaneous lesion that had appeared 48 hours prior to the consultation, slightly painful. He did not report fever, trauma, or intense exercise. He had preserved general state and vital signs: BP 155/90, HR 85/min, basal SpO2 98%, and temperature of 37°C. Physical examination revealed a diffuse erythematous lesion on his left torso, indurated, and poorly delimited (Figure 1). Blood analysis showed C-reactive protein 19 mg/dL, creatine-kinase 88 U/L, creatinine 3.5 mg/dL, hemoglobin 104 g/L, leucocytes 4.62·10^9^/L [neutrophils 4.2·10^9^/L, lymphocytes 0.3·10^9^/L], platelets 224·10^9^/L, and prothrombin time 100%. A CT-scan was performed immediately, which showed thickening of left pectoral muscles and trabeculation of adjacent adipose tissue, without collections or gas (Figure 2). Given the suspicion of an infectious process, empirical treatment was started with ceftazidime and linezolid, and the patient was hospitalized. No microorganisms were isolated in blood cultures. Staphylococcus aureus methicillin-sensitive (MSSA) was isolated in nasal swab. One week later, despite antibiotic treatment, the lesion presented progressive fluctuating consolidation. An MRI was performed then, revealing a pectoral septated collection (59x52x42mm) (Figure 3) and confirming the diagnosis of pyomyositis. No signs of endocarditis as primary process were detected in the echocardiogram. MSSA was isolated in purulent fluid obtained by endoscopic ultrasound fine-needle aspiration. The lesion was surgically debrided and treatment was changed according to the sensitivity of the antibiogram to clindamycin and levofloxacin, for a duration of 4 weeks, achieving complete resolution of the process.

## 3. Discussion

The true incidence of pyomyositis is unknown since it is an underreported disease [1]. It usually occurs in the context of muscle injury, surgery, or ischemia, or also when the host's defense mechanisms are compromised, such as in immunosuppressed patients, diabetes mellitus, renal insufficiency, liver disease, AIDS with low CD4+ lymphocytes count, or use of intravenous drugs [2].* Staphylococcus aureus* (SA) is the main causative agent in up to 85% of cases, but in case of severe cellular immunodeficiency, other agents such as Candida (the main fungal etiology),* Nocardia*,* Mycobacterium*,* Aspergillus*,* Fusarium,* or* Cryptococcus *must be considered [3].

There is a first invasive stage with mild pain, swelling, and variable fever. Initially there are no purulent collections; however, purulent collections occur after 7-14 days [4]. Pyomyositis can mimic other serious infections, such as necrotizing fasciitis and gas gangrene, usually caused by* Streptococcus pyogenes* and* Clostridium perfringens*, respectively. An intense and disproportionate pain or the presence of gas is typical for the latter two entities. Distinguishing them is crucial, given that surgical treatment is more aggressive in fasciitis and gas gangrene, and they have a greater mortality (50% of cases) in comparison with myositis (1-10%). The large muscles of the lower extremities are most commonly affected, such as quadriceps and gluteus [1], being unusual the localization of pyomyositis presented in this patient.

Despite muscle inflammation, muscle enzymes are typical and paradoxically normal. Only 5-35% of blood cultures are positive, whilst cultures of samples obtained from the abscess are positive in almost 100% of the cases [4]. Regarding imaging techniques, the gold-standard is MRI, where it is observed an increase in muscle volume and, in case of abscess, an isointense or hyperintense central zone. Moreover, MRI allows studying involvement of adjacent structures (joints, bone, soft tissue, etc.). CT is more accessible and faster, enabling an early approach to the extension of the lesions, as well as ruling out the presence of gas. It may nonetheless fail to demonstrate inflammatory changes in early stages [5]. Ultrasound is less specific, and radionuclide scans are unable to precisely determine the site of infection, not distinguishing muscular abscess from necrotizing fasciitis or myositis. However, the latter can be useful in cases of multifocal involvement (for instance, septic metastasis), especially in those with documented bacteremia.

In immunosuppressed populations, empirical antibiotics to treat myositis should cover methicillin-resistant SA (MRSA), gram-negative, and anaerobic bacteria. There are many possible combinations, on which antibiotic activity should be assessed against adverse effects. Empirical treatment in our case was ceftazidime and linezolid. If the latter is used, blood count should be monitored closely, since it is a potential myelosuppressor. Other options for MRSA would be daptomyicin (taking into account its potential muscle toxicity), clindamycin, or vancomycin (potentially nephrotoxic). Clindamycin, in addition, has activity against anaerobes, frequently involved in abscesses. Ceftazoline could be useful as second-line for MRSA and gram-negative [6], although it is also myelosuppressor. However, in addition of antibiotic treatment, surgical debridement in case of abscess is imperative. In fact, in this case, despite treatment with linezolid, improvement was only observed after debriding the lesion.

Immunosuppression should be considered as a predisposing factor in this patient. Our patient was on everolimus and prednisone. Although mTOR inhibitors have been associated with delayed tissue regeneration [7, 8], no comparative studies are available with other immunosuppressants such as calcineurin inhibitors or mycophenolate. From our point of view, it seems advisable to temporarily reduce immunosuppression in these patients to a minimum during the acute phase of infection taking into account the severity of the infection and the immunological risk of the patients.
